# 3D deep convolutional neural network segmentation model for precipitate and porosity identification in synchrotron X-ray tomograms

**DOI:** 10.1107/S1600577522006816

**Published:** 2022-07-29

**Authors:** S. Gaudez, M. Ben Haj Slama, A. Kaestner, M. V. Upadhyay

**Affiliations:** a Laboratoire de Mécanique des Solides (LMS), CNRS UMR 7649, Ecole Polytechnique, Institut Polytechnique de Paris, Route de Saclay, 91128 Palaiseau Cedex, France; b Laboratoire de Mécanique des Sols, Structures et Matériaux (MSSMat), CNRS UMR 8579, CentraleSupélec, Université Paris-Saclay, 91190 Gif-sur-Yvette, France; c Laboratory for Neutron Scattering and Imaging, Paul Scherrer Institut, CH-5232 Villigen-PSI, Switzerland; Paul Scherrer Institut, Switzerland

**Keywords:** 3D U-net, deep convolutional neural network, TXM, nanotomography, additive manufacturing

## Abstract

New developments at synchrotron beamlines and the ongoing upgrades of synchrotron facilities allow the possibility to study complex structures with a much better spatial and temporal resolution than ever before. However, the downside is that the data collected are also significantly larger (more than several terabytes) than ever before, and post-processing and analyzing these data is very challenging to perform manually. This issue can be solved by employing automated methods such as machine learning, which show significantly improved performance in data processing and image segmentation than manual methods. In this work, a 3D U-net deep convolutional neural network (DCNN) model with four layers and base-8 characteristic features has been developed to segment precipitates and porosities in synchrotron transmission X-ray micrograms. Transmission X-ray microscopy experiments were conducted on micropillars prepared from additively manufactured 316L steel to evaluate precipitate information. After training the 3D U-net DCNN model, it was used on unseen data and the prediction was compared with manual segmentation. A good agreement was found between both segmentations. An ablation study was performed and revealed that the proposed model showed better statistics than other models with lower numbers of layers and/or characteristic features. The proposed model is able to segment several hundreds of gigabytes of data in a few minutes and could be applied to other materials and tomography techniques. The code and the fitted weights are made available with this paper for any interested researcher to use for their needs (https://github.com/manasvupadhyay/erc-gamma-3D-DCNN).

## Abstract 2

A 3D U-net deep convolutional neural network has been developed and tested to segment precipitates in synchrotron-based X-ray tomography experiments. Comparison of predicted segmentation showed a good agreement with manual segmentation.

## 1. Introduction

Synchrotron X-ray tomography is a non-destructive characterization technique that allows distinguishing microstructural features exhibiting different X-ray attenuation contrasts (absorption or phase) in three dimensions (3D). This technique is typically used to investigate phase changes as well as the morphology, position and distribution of chemical heterogeneities occurring at different length scales in different material systems (Withers *et al.*, 2021 ▸). In alloys, the technique is widely used to investigate porosities (Dinda *et al.*, 2016 ▸), precipitates (Kaira *et al.*, 2018 ▸; Torbati-Sarraf *et al.*, 2021 ▸) and phase changes (*e.g.* liquid to solid) (Daudin *et al.*, 2017 ▸).

Synchrotron X-ray tomography experiments typically generate a large amount of data (several terabytes), especially with the development of time-resolved tomography (García-Moreno *et al.*, 2021 ▸), and quantitatively analyzing these data is often very challenging. One of the main challenges in data analysis is to perform an unambiguous segmentation of the features of interest. The presence of (i) multiple kinds of features of interest exhibiting similar X-ray absorption coefficients and (ii) background noise with a brightness contrast that is similar to some small features of interest can make it very difficult to segment the data using conventional one-parameter techniques, *e.g.* manual gray-scale thresholding, Otsu’s method (Otsu, 1979 ▸), *etc*. While filters can be used to enhance the contrast between features and background noise in order to facilitate such segmentation (Kaestner *et al.*, 2008 ▸), nevertheless, conventional single-parameter gray-scale-based techniques remain highly limited in their predictive capabilities. For example, they are unable to account for the 3D nature of features such as feature size and morphology, which can play an important role in distinguishing between different kinds of features.

One solution could be to perform manual segmentation. However, this approach is highly time consuming and requires a high attention to detail, which is impractical when dealing with large amounts of data. In addition, manual segmentation suffers from human bias, which varies from one operator to another.

A more practical, and at times more accurate, solution is to automate the segmentation process using deep learning methods (LeCun *et al.*, 2015 ▸). Deep learning is a subset of machine learning methods that eliminates data pre-processing involved in machine learning methods by using unstructured data (unorganized data, *e.g.* an image) as input instead of structured data (organized and tabulated data, *e.g.* a phone book). Deep learning methods are composed of a succession of operations aimed at finding data patterns and extracting useful feature information. They are widely used to process images, in particular to detect objects (object detection) and recognize differences between them (object recognition), to characterize an entire image (scene summarization), and to associate each pixel of an image with a class of objects present in the image (semantic segmentation) in many different fields including medical, engineering and self-driving vehicles.

The convolutional neural network (CNN) is a subset of deep neural network methods, commonly used for image analysis. The difference between a CNN and a neural network is the use of the convolution operation (convolutional layer) allowing the former to capture local features as well as reduce the complexity of the network. Semantic segmentation is one of its applications. This approach significantly improves the segmentation quality with respect to the single-parameter gray-scale-based techniques for detection of microstructural features such as porosities (Gobert *et al.*, 2020 ▸), precipitates (Senanayake & Carter, 2020 ▸; Torbati-Sarraf *et al.*, 2021 ▸), and phase and grain boundaries (Furat *et al.*, 2019 ▸; DeCost *et al.*, 2019 ▸; Ajioka *et al.*, 2020 ▸). In addition, it allows simultaneous segmentation of objects such as precipitates (Senanayake & Carter, 2020 ▸; Torbati-Sarraf *et al.*, 2021 ▸), and phase and grain boundaries (DeCost *et al.*, 2019 ▸; Ajioka *et al.*, 2020 ▸). Originally proposed by Ronneberger *et al.* (2015 ▸) for semantic segmentation of bio-medical images, U-Net is a special type of deep CNN (DCNN) architecture for image segmentation. Thus far, DCNN-based segmentation of data collected from tomography experiments has been carried out by slicing volumes into 2D slices and performing independent segmentation of each slice (Furat *et al.*, 2019 ▸; Gobert *et al.*, 2020 ▸; Torbati-Sarraf *et al.*, 2021 ▸; Bellens *et al.*, 2021 ▸; Ali *et al.*, 2021 ▸). In following this approach, however, the 3D information of the features such as their size and morphology is lost. Then, in order to recover the size and morphology information, one must perform a 3D reconstruction of the segmented slices. However, the error incurred in first slicing the data, segmenting the 2D slices and then reconstructing the segmented data is higher than directly segmenting the 3D data set. To meet the needs of volumetric data segmentation, Çiçek *et al.* (2016 ▸) proposed a 3D U-Net architecture showing a performance gain to an equivalent 2D architecture. An example of its application in material science can be found in the recent work by Furat *et al.* (2019 ▸) who compared DCNN-based segmentation of grains using both the aforementioned techniques on data collected from 3D X-ray diffraction experiments on an Al alloy. Their comparison revealed that the model was able to segment better when it was trained directly on 3D data.

In this work, we have numerically implemented a slightly modified version of an existing 3D DCNN based on the U-net architecture (Çiçek *et al.*, 2016 ▸) in Python. The differences between the two approaches are explained in Section 3. The modified 3D U-net DCNN model is designed to segment sphere-shaped features such as precipitates and porosities in 3D data-sets. The model is applied for the first time to identify submicrometre-sized precipitates and porosities where both objects and background present similar brightness contrast from transmission synchrotron X-ray microscopy (TXM), or nanotomography experiments conducted on micropillars extracted from laser metal deposited (LMD, an additive manufacturing technique) 316L stainless steel (316LSS) subjected to thermal treatments. The proposed model is found to be significantly faster than manual segmentation and it is able to automate segmentation of investigated objects while preserving the high-resolution details. Finally, along with this paper, the entire code and the fitted weights have been made available for any interested researcher to use to segment similar data-sets in order to avoid the significantly time-consuming part of data preparation and training.

## 2. Experimental procedure

### 2.1. Material and sample preparation

The 316LSS powder used in this work to additively manufactured samples was prepared using the inert gas atomization process by Höganäs AB. The wrought alloy used to manufacture the powder had the following chemical composition (wt%):

Fe-16.9Cr-12.7Ni-2.5Mo-1.5Mn-0.7Si-0.015P-0.011C-0.005S.

Additive manufacturing was performed via the LMD process using the ‘Mobile’ machine from BeAM (AddUp). A single-track bidirectionally printed three-layer thin-wall of dimensions 100 mm × 0.6 mm × 0.6 mm (*x*, *y*, *z*) was manufactured using this machine; the process parameters were: laser power = 225 W, powder flow rate = 6.5 g min^−1^, deposition speed = 2000 mm min^−1^, vertical displacement of focusing head = 0.2 mm after depositing one layer. Additional details on the material, powder characteristics and specifications of the LMD machine have been given by Upadhyay *et al.* (2021 ▸) and Ben Haj Slama *et al.* (2022 ▸).

The as-built wall and substrate had been cut near the mid-section and along the direction normal to the build and print directions (*yz* plane). This cross-section had been mechanically polished first using SiC papers with different grits (from 800-grit to 4000-grit) followed by diamond paste polishing with grit sizes 3 µm and 1 µm and finally ion polishing. Scanning electron microscopy (SEM) and electron back-scattered diffraction analysis were performed on these samples using an Environmental-SEM Quanta 650 FEG microscope to identify regions of interest for micropillar extraction. Cylindrical micropillars ∼25 µm in diameter and ∼75 µm in height were extracted from the second and third deposited layers using focused ion-beam (FIB) milling inside an FEI Helios Nanolab 660 dual-beam scanning electron microscope, which is equipped with a dual-beam FIB. Then, the micropillars were subjected to different solid-state thermal cycling (SSTC) to mimic the thermal cycling that would occur during additive manufacturing but under controlled conditions that allow us to monitor the evolution of sub-micrometre-sized precipitates and porosities in the bulk material. These thermal treatments were performed inside an FEI Titan3 G2 60-300 transmission electron microscope (TEM). Each micropillar was attached to an electrothermal chip, which can be inserted into an electrothermal TEM sample holder (Protochips). This sample holder allows SSTC to be performed inside the controlled environment of the Titan3 TEM. Further information about the technique used for applied controlled SSTC is given by Ben Haj Slama *et al.* (2022 ▸). The micropillars were characterized via TXM before and after each SSTC performed inside the TEM.

### 2.2. TXM

TXM experiments were conducted on micropillars at the ANATOMIX beamline of the SOLEIL synchrotron (Gif-sur-Yvette, France). A monochromatic X-ray beam of energy 16.87 keV was used for the acquisitions allowing a small intensity attenuation (∼40%) according to the sample thickness of the Fe-based micropillars (∼25 µm). The condenser, which illuminated the samples, was placed at 2 m in front of the sample. The objective zone plate was 69.68 mm from the sample. The Hamamatsu C12849-112U detector was placed at 30 m from the sample leading to a pixel size resolution of 29.33 nm (using 2 × 2 binning). TXM experiments were performed by acquiring 1000 projections over an angular range of 180° at room temperature. For each sample, the acquisition was repeated four times and each set of four acquisitions were merged to improve the signal-to-noise ratio. Data pre-processing and tomographic reconstruction were performed with the *PyHST* software package. The tomography color scale is a gray scale from black to white: highly dense regions (*i.e.* steel matrix) appear in bright contrast and low-density regions (*i.e.* precipitates and environment) appear in dark contrast.

## 3. 3D neural network-based segmentation model

### 3.1. CNN

We have based our 3D U-net DCNN approach on the work of Çiçek *et al.* (2016 ▸) to perform semantic segmentation of precipitates from TXM experiments performed on micropillars extracted from the LMD 316LSS thin-wall. The key difference between our model and that of Çiçek *et al.* (2016 ▸) is that our model doubles the number of channels after the max pooling operations instead of before. In addition, our model uses a smaller number of characteristic features per layer. These modifications allow us to decrease the number of training parameters and thus speed up training and prediction while still maintaining a good accuracy.

**Figure 1:**
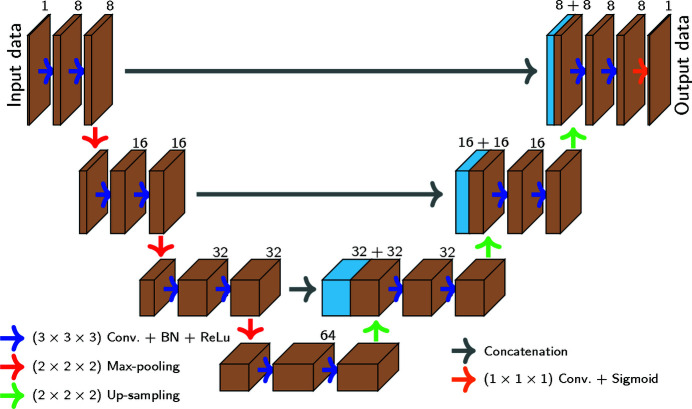
The 3D U-net DCNN used in the present work has four layers and base-8 characteristic features. Arrows represent the different operations and brown and blue boxes represent the multi-channel and copied feature volumes, respectively. The input and output volumes are gray-scale data with the same pixel size resolution.

The architecture of our 3D U-Net DCNN is presented in Fig. 1 ▸ and the layer operations are presented below; definitions of the different layer operations described below are given in Appendix *A* [App appa]. The 3D U-net DCNN model consists of two main parts: a contracting part (left) and an expanding part (right). The contracting part consists of repeated units: two convolutions with a (3 × 3 × 3) kernel size, each followed by a batch-normalization (BN) and the rectified linear unit (ReLU) activation function and finally by a max pooling operation with a (2 × 2 × 2) kernel size with a stride of two in each dimension. The sequence is repeated twice. The expanding part consists of repeated units: up-sampling with a (2 × 2 × 2) kernel size with a stride of two in each dimension followed by a concatenation with the corresponding feature map from the contracting part and finally two convolutions with a (3 × 3 × 3) kernel size. Each convolution is followed by a BN and the ReLU activation function. To keep the symmetry and restore a higher-resolution image, this sequence is also repeated twice. The final layer consists of one convolution with a (1 × 1 × 1) kernel size and a sigmoid activation function providing the segmented map. The sigmoid activation function allows to normalize the output [0:1] returning the probability of each pixel belonging to a class. The output image has the same size resolution as the input image by adding extra rows and columns around the feature maps for each convolution (*i.e.* same padding). Direct links between the contracting and expanding parts (concatenation) allow to avoid bottleneck problems and loss of information by combining higher-resolution maps from the contracting to the expanding part. The concatenations allow the spatial information to be preserved during image reconstruction.

Previous investigations via TEM (Upadhyay *et al.*, 2021 ▸; Ben Haj Slama *et al.*, 2022 ▸) have revealed the presence of precipitates (oxide, non-oxide and mixed precipitates) at a length scale that is similar to the objects (precipitates or porosities) observed in TXM micrographs, although no distinction can be made between the objects in the TXM micrographs. Furthermore, while no porosities were observed in the TEM investigations (Upadhyay *et al.*, 2021 ▸; Ben Haj Slama *et al.*, 2022 ▸), it has to be reminded that TEM investigations only probe a very small volume within which porosities may not have been present. In comparison with TEM lamellae, micropillars have a volume that is four orders of magnitude higher. As no distinction can be made between precipitates and porosities by TXM, the present problem simplified to the classification on each pixel between two classes: the objects and the background, which is a binary task. Following this, the performance of the 3D U-net DCNN is calculated with the binary cross entropy loss function. This function evaluates the difference between the pixel class, which is known, and the probability calculated by the model to be in the corresponding class during the training session. The loss function is based on a negative logarithm allowing to strongly penalize the predictions having a low probability (*i.e.* bad predictions). The Adam optimizer (Kingma & Ba, 2014 ▸) is used to reduce the loss value by updating the network parameters at each epoch (*i.e.* one cycle through the full training dataset). The proposed 3D U-net DCNN model has four layers and base-8 characteristic features which result in 366593 parameters. Out of these parameters, 365889 are trainable corresponding to the weights, kernel filters and bias, and 704 are non-trainable corresponding to BN.

The 3D U-net DCNN is implemented in Python version 3.8.5 using the Keras Python library version 2.4.3 and the Tensorflow library version 2.5.0. To speed up the computations, the code was executed on a graphics processing unit (GPU) from NVIDIA. To that end, Cuda version 11.2 and cuDNN library version 8.2.1 were used. The training, validation and prediction were performed with the following hardware specifications: NVIDIA Quadro RTX 5000 GPU, Intel(R) Xeon(R) Gold 5220R 2.2 GHz 48-core CPU, 384 GB RAM and Ubuntu 20.04 desktop operating system.

### 3.2. Data preparation

**Figure 2:**
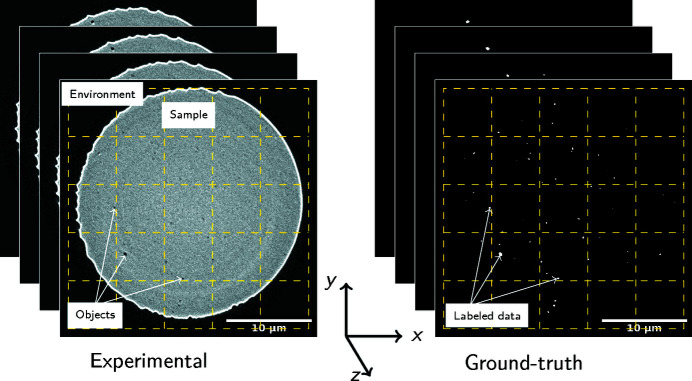
TXM synchrotron experimental result and ground-truth (manually segmented) images with 1024 × 1024 × *z* voxels from a ∼25 µm-diameter micropillar extracted from an additively manufactured 316L stainless steel sample (*z* represents the number of slices along its direction, *e.g.* four in the figure). Dashed yellow squares represent the 192 × 192 × 192 voxels sub-volumes used to train the neural network.

Training the model requires two types of volumes, which are shown in Fig. 2 ▸ – the experimental TXM gray-scale data obtained after reconstruction and the ground-truth data. In the experimental data, the bright contrast (disk) represents a slice of the micropillar while the surrounding dark contrast is the environment. Dark spots within the disk are objects of interest (precipitates or porosities or both). These objects have been manually segmented to obtain the ground-truth data. The bright ring encircling the disk is an undesirable artifact due to the TXM technique.

**Figure 3:**
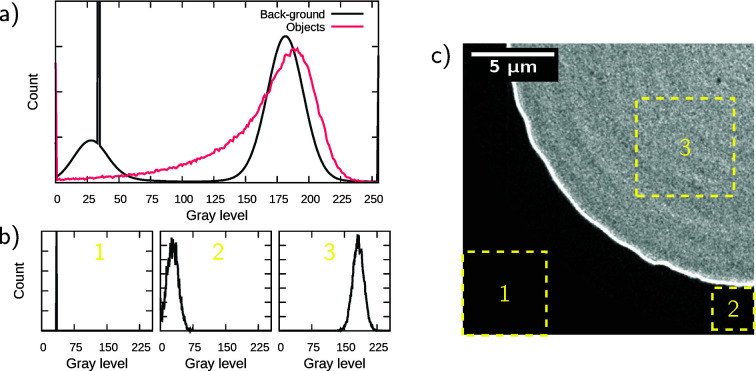
(*a*) Pixel intensities of background and objects (both features have their own intensity scales). (*b*) Pixel intensities of the corresponding volumes numbered in the TXM partial image. (*c*) Partial TXM image. Pixel intensity analysis was performed on the volume data.

Fig. 3 ▸(*a*) shows the gray intensity histograms of the background and the objects. Due to the very small object fraction the area under the black curve (background) represents ∼99.7% of the voxels while the area under the red curve represents the remaining ∼0.3%, thus the two histograms have different intensity scales allowing better visualization. Fig. 3 ▸(*b*) shows gray intensity histograms corresponding to the yellow squares shown in Fig. 3 ▸(*c*). They allow us to understand the background histogram (here background histogram refers to all the pixels that are not the investigated objects, *i.e.* environment and sample): the sharp peak at the gray-scale value of 34 is part of the region outside of the reconstruction, the broad peak in the gray-scale range 0–75 is sample environment near to the sample, and the broad peak in the gray-scale range ∼125–225 is the sample. For area 3 in Figs. 3 ▸(*b*) and 3(*c*), the investigated objects are accounted for but they have a low volume fraction, which makes it difficult to deduce their presence. The overlap of gray-scale intensities between the objects and the matrix highlights the difficulties to quantitatively segment such objects using a one-parameter gray-scale-based manual thresholding and segmentation approach (*e.g.* filters and mathematical operations) and strongly supports the development of a machine learning method.

The input data for training the 3D U-net DCNN model was prepared in the following manner. Experimental data were normalized with a Z-score normalization (*i.e.* zero mean and unit variance) process allowing to standardize the input and increase the machine learning performance. Labeled data were obtained by manually labeling the objects of each slice of the volume using *ImageJ* software (Schindelin *et al.*, 2012 ▸) and then 3D merging using the merge channel function in this software. Two operators separately performed the manual labeling for training, validation and test data in order to limit human bias. Manually marking the objects is fastidious and time-consuming work but it allows better results to be obtained than the single-parameter gray-scale-based segmentation approach and justifies the use of a neural network. It has to be mentioned that few small objects were missed by the operators during manual segmentation as will be highlighted in the *Results* [Sec sec4] section. The ground-truth data could also be generated from segmentation recipes (*e.g.* sequence of filters, segmentation, erosion and reconstruction), as in Gobert *et al.* (2020 ▸), but the resulting set of fitted parameters for the neural network to be trained on will not lead to better segmentation than the recipes themselves.

### 3.3. Neural network training, validation and testing

To train the 3D U-net DCNN, the original data were divided into non-overlapping sub-volumes of 192 × 192 × 192 voxels (multiple of 8). The sub-volumes are illustrated in Fig. 2 ▸ by dashed yellow lines. A batch size of only two sub-volumes could be used due to GPU memory limitation. Three different samples were used: two samples for training and validation data and one sample for testing. In total 1280 slices from three micro-pillars were used: 60% of these slices were used for training, 30% for validation and 10% for testing. Finally, to limit over-fitting and generalize the 3D U-net DCNN, up-scaling operations were used.

## 4. Results

For the present study, background class (*i.e.* sample matrix and environment) represents more than 99.8% of the volume of the test data set. A measure of the global accuracy is often used to characterize the quality of segmentation models (Ajioka *et al.*, 2020 ▸; Senanayake & Carter, 2020 ▸; Ali *et al.*, 2021 ▸). Here, due to the very low volume of the objects investigated, a measure of the global accuracy is not relevant. For example, if the whole volume is predicted as background, then the accuracy will be more than 99.8% even though no object is correctly segmented.

**Figure 4:**
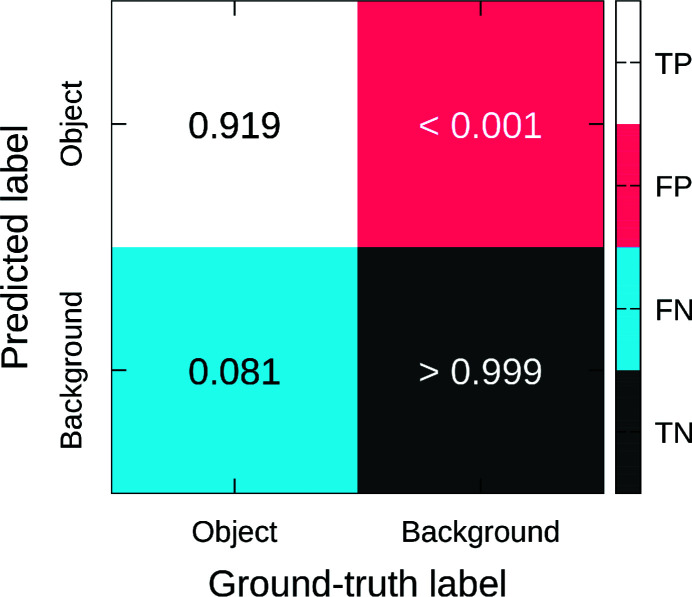
Normalized confusion matrix for the test set. TP: true positive; FP: false positive; FN: false negative; TN: true negative.

Predicted outputs by the DCNN are gray-level matrices in which the gray-levels represent the probability to be within a class: gray-level 0 is background class and gray-level 255 is objects class. A probability of 0.5 (gray-level 127) was used as a threshold between the two classes during training and validation, *i.e.* pixels with a gray-level equal to or lower than 127 are background and pixels with a gray-level higher than 127 are objects. Thus, predicted outputs were thresholded at 127 to compare with ground-truth data. Fig. 4 ▸ shows the confusion matrix (Fawcett, 2006 ▸) obtained from a pixel-by-pixel comparison of the ground-truth and predicted data for the test set. This representation allows to highlight the accuracy of the segmentation for each class (objects and background), independently. Background prediction has the highest accuracy (error lower than 0.001). Object prediction has a good accuracy (error of 0.081, *i.e.* 8.1% of pixels belonging to the object class from the ground-truth data are predicted as belonging to the background class); recall that objects represent less than 0.2% of the volume.

**Figure 5:**
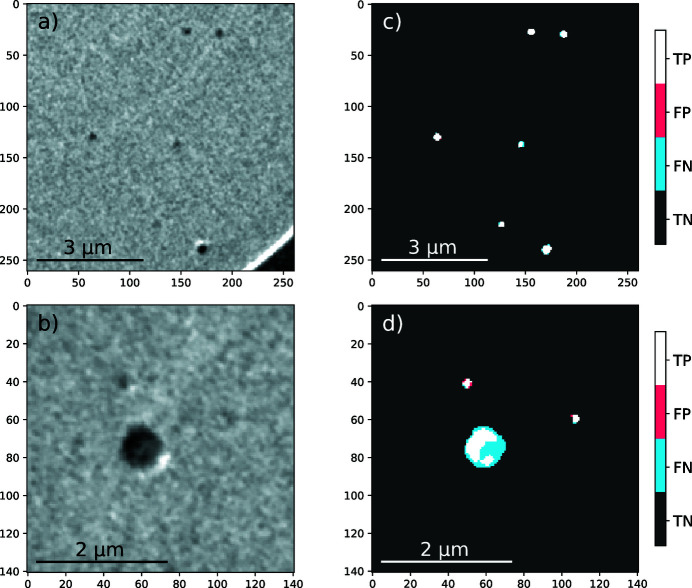
TXM raw data (*a* and *b*) and hit map (*c* and *d*). TP: true positive; FP: false positive; FN: false negative; TN: true negative.

Fig. 5 ▸ shows qualitative examples of segmentation and overlaying between ground-truth and predicted data. Figs. 5 ▸(*a*) and 5(*b*) show reduced windows of TXM raw data. In Figs. 5 ▸(*c*) and 5(*d*), white areas are true positive, black areas are true negative, red areas are false positive and blue areas are false negative predictions. True positive or negative predictions are pixels correctly predicted according to the ground-truth while false positive or negative are not. In general, a good agreement is found between the ground-truth and the predicted data [Fig. 5 ▸(*c*)]. However, the model tends to underestimate the object size in comparison with manual segmentation. Fig. 5 ▸(*d*) shows an example of a bad prediction by the model because of the size of the object, which is much larger than other ones [*e.g.* as seen in Fig. 5 ▸(*c*)]. This bad prediction is explained by the training and validation set in which such object sizes are sparse; *e.g.* for the test set, there are only two large objects that are badly segmented out of 666. Due to their small number, the model was not able to learn such features (even with the up-scaling operations). Only two examples are given in the present work, but raw data, ground-truth data and predicted data of the test set are made available as supporting information.

**Figure 6:**
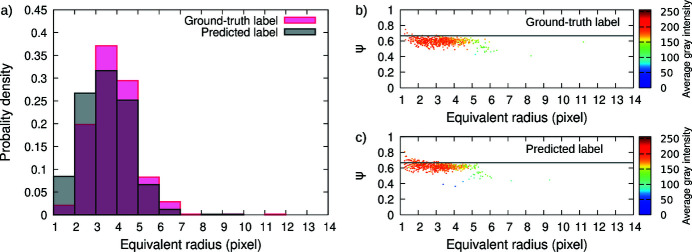
(*a*) Histogram of the object size distribution from ground-truth data and predicted data. (*b* and *c*) The sphericity (ψ) parameter as a function of radius for the ground-truth and predicted data, respectively, for the test set. Color scales in (*b*) and (*c*) represent the average gray intensity value of the objects. The gray line at ψ = 2/3 is the parameter ψ for a sphere discretized by voxels without surface reconstruction.

Fig. 6 ▸(*a*) shows the size distribution histograms comparing the ground-truth data and the predicted data from the test set. Table 1 ▸ shows quantitative information about objects. Because the model accounts for 3D information, objects at the surface of the investigated volume (*i.e.* objects in contact with the first and last slice in all directions; *e.g.* *z* = 0 and *z* = 128) are not well segmented and were excluded from the quantitative analysis. In addition, objects with a volume lower than 8 voxels (2 × 2 × 2 voxels) are considered to be below the experimental resolution limit and they were also excluded. As qualitatively observed, prediction slightly underestimates the object size in comparison with the ground-truth data (the mean radius is 3.34 and 3.02 pixels for the ground-truth and predicted data, respectively). But a higher density of objects is found by the model (difference of 105 objects) and they mostly fall within the 1–3 pixel radius, thus decreasing the mean radius measured. Most of the additional objects found by the 3D U-net DCNN are objects missed by the operators during manual segmentation.

Figs. 6 ▸(*b*) and 6(*c*) show the measure of the sphericity as a function of the equivalent radius as well as the average gray intensity of the objects. The sphericity parameter characterizes the degree of spherical shape of the object, and reads (Wadell, 1932 ▸)

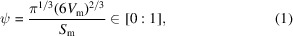

where *V*_m_ is the measured volume and *S*_m_ is the measured surface of the object. ψ = 1 for a perfect sphere and it decreases with the increase of the degree of non-spherical shape of the object. However, in the absence of surface reconstruction (*i.e.* polygonal meshing) (Lorensen & Cline, 1987 ▸; Rorato *et al.*, 2019 ▸), due to the cubic shape (voxel) discretization of the volume, the sphericity parameter is biased. For a sphere with a cubic shape discretization, the sphericity parameter tends toward ∼2/3 (*i.e.* for a cubic shape discretization volume, 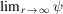 ≃ 2/3 where *r* is the radius; see the supporting information).

Results show that the objects investigated are close to a sphere-like morphology as qualitatively observed and that smaller objects have a higher average gray intensity than bigger objects. Both observations are accurately captured by the 3D DCNN model.

Finally, to test the significance of the proposed model for object segmentation, 3D U-net DCNN models with different configurations were trained and evaluated on the same set of data. Due to hardware limitations, only configurations with lower numbers of layers as well as lower base numbers of characteristic features could be tested. To be comparable, each model was trained over 240 epochs and evaluated with class­ification statistics (Fawcett, 2006 ▸). Metrics used to quantify and compare model performances are

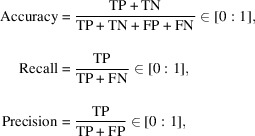

where TP means true positive, FP means false positive, FN means false negative, and TN means true negative. The valid range of the accuracy is very limited due to the class imbalance. Table 2 ▸ shows statistics for each trained model after 240 epochs. The originally proposed model with four layers and base number of eight characteristic features has the best statistics over others. The model with three layers and a base number of eight characteristic features also presents good statistics with a reduced number of parameters allowing to be used with a less powerful hardware specification, but training computation time remains similar. For a given model, in general, decreasing its number of layers or base number of characteristic features leads to less accurate predictions. However, the model with four layers and base number of six characteristic features is found to perform worse than the four layers and base number of four characteristic features. Finally, the computation time per epoch is not linearly proportional to the number of parameters of the model and the largest gains are obtained by decreasing the base number of characteristic features, but this adversely affects the accuracy of the segmentation.

## 5. Discussion

The proposed machine learning algorithm is able to segment small objects well with a very small volume fraction and with gray-scale intensities similar to the background from TXM data. Qualitative and quantitative comparisons between the ground-truth and predicted data gave good results. In addition, the segmentation quality is significantly improved in comparison with single-parameter gray-scale thresholding. However, it was also shown that some large objects may not be correctly segmented by the model because of their small density in the training and validation sets. A radius of ∼8 pixels (*V* ≃ 2000 voxels) can be seen as an upper limit of the present fitted weights and model (see supporting information). In the case where only large objects are present, the resolution of the data can be decreased to eliminate this issue.

Our 3D U-net DCNN model is able to segment a volume of 1024 × 1024 × 256 voxels in 1 min 32 s with the hardware specifications (given in Section 3.1) without human intervention. In comparison, two humans took ∼60 h in total to segment one data-set of the same size. These numbers show the significant time gains with high accuracy that can be achieved when using machine learning models to treat large amounts of data without any human intervention after the model has been trained. The proposed model showed the best performance over other lighter configurations and implementations where the base number of characteristic features and layers were decreased. However, the time to segment the aforementioned volume is slightly longer than using a model with fewer parameters (*e.g.* 1 min 22 s for model three layers and base number of four characteristic features).

The main issue of the development of a machine learning algorithm is data preparation for training and validation. The preparation is fastidious, time-consuming and without guarantee of success. For this purpose, the fitted weights as well as the Python codes used in the present work are shared (see supporting information). They can be used by researchers facing similar problems as discussed in the present study to segment data or make tests before preparing their own model or data or both. Furthermore, simulated data can be used to train the model and to facilitate machine learning (Ali *et al.*, 2021 ▸).

## 6. Conclusions

In the present work, a 3D U-net DCNN model was developed to segment precipitates and porosities from micrograms obtained from synchrotron-based TXM experiments performed on micropillars extracted from an additively manufactured 316L stainless steel. The 3D U-net DCNN architecture was used to improve the segmentation of the volumetric data, to speed up and automate analysis, and to limit human intervention. After training and validation, the model was applied to an unseen data-set to test its robustness. Quantitative (confusion matrix and histogram of sizes) and qualitative (visual assessment of the segmentation) investigations were used to interpret the results. They showed that the 3D U-net DCNN architecture is able to segment investigated objects and preserve the high-resolution details. In this study, the predicted data are even better than the ground-truth because of objects missed by the operators during manual segmentation but predicted by the DCNN. The proposed model showed the best statistics and the fastest training rate over others tested, while it has the largest number of parameters. However, it has the longest training and predicting times because of its larger number of parameters. Although developed and applied to an additively manufactured 316L stainless steel investigated by TXM, the proposed model and fitted weights could be used for different materials and tomography experiments as long as the data share common features with the data used in this work. In order to facilitate other users in their studies, the entire code and the fitted weights of the proposed model is provided (see https://github.com/manasvupadhyay/erc-gamma-3D-DCNN).

## Supplementary Material

Sections S1 ansd S2. Table S1. Figure S1. DOI: 10.1107/S1600577522006816/gy5036sup1.pdf

## Figures and Tables

**Table 1:** Quantitative object information for the ground-truth data and the predicted data for the test set

Data	Number of objects	Mean radius (pixel)	Standard deviation (pixel)	Sphericity
Ground-truth	666	3.34	1.06	0.595
Predicted	771	3.02	1.09	0.622

**Table 2:** Object segmentation performance metrics for different 3D U-net DCNN models (L: layers; CF: characteristic features) configuration and implementation after 240 epochs; absolute time per epoch is also provided and it is specific to the software and hardware specifications used in the present work

Model	Number of parameters	Min/epoch	Accuracy	Recall	Precision
4 L/base-8 CF	365593	∼4.48	0.999334	0.696546	0.845458
3 L/base-8 CF	89153	∼4.42	0.999222	0.659309	0.802174
4 L/base-6 CF	206581	∼3.02	0.998942	0.572835	0.689927
3 L/base-6 CF	50341	∼2.88	0.998555	0.478548	0.536446
4 L/base-4 CF	92145	∼2.15	0.999086	0.627773	0.741001
3 L/base-4 CF	22545	∼2.03	0.998885	0.436197	0.734485

## Appendix A Definitions of the different layer operations

*Convolution operation* consists of applying a filter (kernel weights) to an area and shifting and repeating the process over the input data. At each slide, the kernel weights are multiplied by the part of the input data being treated. Making the sum of this multiplication leads to defining a single pixel value in the feature map. This operation allows extracting features present in the input such as lines.

*Pooling operation* consists of retaining the most important features (summarize) from each subregion (information best describing the image context) of the feature map leading to a reduction in its size and have fewer trainable parameters. Summarizing can be performed with different functions, but the most common use is max pooling in which only the maximal value of the subregion is kept.

*Activation operation* consists of deciding whether a neuron should be activated or not. It applies a function on values returned by the convolution operation before sending it to the next layer. These functions control how the neural network model learns the training data and the type of prediction the model can make (*e.g.* probability). The most commonly used activation function for hidden layers is the ReLU (rectified linear unit) activation function; this function either returns the input (if the input is greater than 0) or it returns 0 (if the input is less than or equal to 0).

*Up-sampling operation* consists of increasing the size of a feature map. In the simplest version, the operation simply repeats the rows and columns by given factors (*e.g.* 2).

*Batch-normalization* consists of rescaling data to a mean and a standard deviation close to zero and one, respectively. It standardizes the inputs to a layer, stabilizing the learning. The model is then more stable, less sensitive to weight initialization, less prone to overfitting and allows a faster learning rate to be used.
