# Supplementary material for: 3D deep convolutional neural network segmentation model for precipitate and porosity identification in synchrotron X-ray tomograms
Source: J Synchrotron Radiat. 2022 Jul 29;29(Pt 5):1232–40. doi: 10.1107/S1600577522006816 (PMC9455210; doi:10.1107/S1600577522006816)
Supplement: Supplementary file 1 [file s-29-01232-sup1.pdf]

JOURNAL OF SYNCHROTRON RADIATION

Volume 29 (2022)

Supporting information for article:

3D deep convolutional neural network segmentation model for precipitates and porosities identification in synchrotron X-ray tomograms

S. Gaudez, M. Ben Haj Slama, A. Kaestner and M. V. Upadhyay

1 Sphericity ψ parameter

Rounded object morphology can be characterized by the sphericity parameter that gives information about the degree of spherical shape of the object. The sphericity parameter reads [1]:

$$\psi = \frac{\pi^{1/3}(6V_m)^{2/3}}{S_m} \in [0:1] \tag{1}$$

where V_m is the measured volume and S_m the measured surface of the object. $\psi = 1$ for a perfect sphere while it decreases with the increase of the degree of non-sperical shape of the object. However, in absence of surface reconstruction (i.e., polygonal meshing) [2, 3] due to the cubic-shape (voxel) discretization of the volume the sphericity parameter is biased.

To characterize this bias, spheres with different radius were simulated with the sphere equation using the following Python code :

```
import numpy as np
import nrrd

radius = 100 # define the radius of the output sphere

N = int((radius+2)*2)
volume = np.zeros((N,N,N))
Xcenter=Ycenter=Zcenter=N/2

for i in range(N):
    for j in range(N):
        for k in range(N):
            if ((i-Xcenter)**2+(j-Ycenter)**2+(k-Zcenter)**2<=radius**2):
                volume[i,j,k]=1
nrrd.write('radius_'+str(radius)+'.nrrd', volume)
```

The Python code takes as input the radius of the desired sphere and returns a cubic-shape volume containing the sphere. Then, the sphere of each volume was investigated with the *3D object counter* plugin of the ImageJ software [4] returning, among others, the measured volume (V_m) and the measured surface (S_m) of the sphere. The results and calculated sphericity parameters are presented in Table 1 and Figure 1. The results show that:

- Radii calculated from the measured volumes are in good agreement with initial radii and associated error decreases with the radius increase.
- Sphericity ψ parameter tends toward 2/3 with the increase of the initial radius.

Table 1: Initial radius r_0, measured volume V_m, measured surface S_m, sphericity ψ parameter and radius calculated from the measured volume $r(V_m)$ results.

r_0 (pixel)	V_m (pixel^3)	S_m (pixel^2)	ψ	$r(V_m)$ (pixel)
1	7	30	0.5899	1.19
2	33	78	0.6379	1.99
3	123	174	0.6874	3.09
4	257	294	0.6649	3.94
5	515	486	0.6393	4.97
6	925	678	0.6771	6.04
7	1419	894	0.6831	6.97
8	2109	1182	0.6728	7.96
9	3071	1518	0.6731	9.02
10	4169	1902	0.6586	9.98
11	5575	2262	0.6722	11.00
12	7153	2646	0.6785	11.95
13	9171	3174	0.6676	12.99
14	11513	3678	0.6704	14.01
15	14147	4254	0.6650	15.00
25	65267	11766	0.6663	24.98
50	523305	47070	0.6672	49.99
100	4187857	188502	0.6665	99.99
200	33507884	753774	0.6668	200.00
500	523592064	4712094	0.6667	500.00

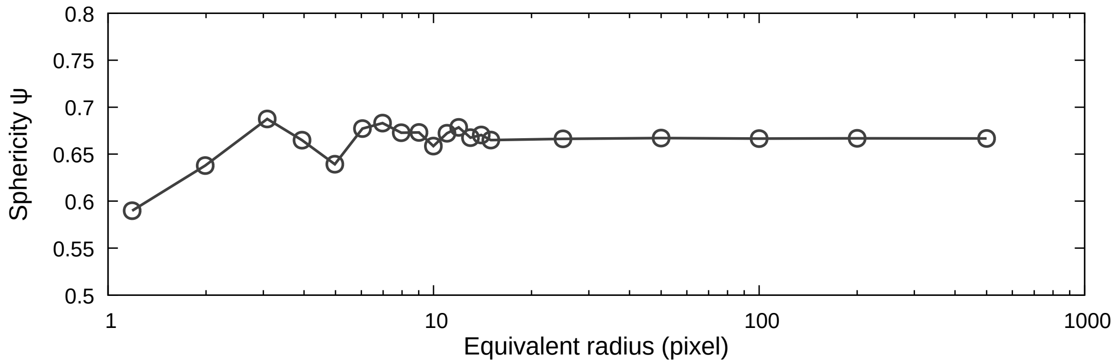

Figure 1: Sphericity ψ parameter as function of the recalculated radius from the measured volume for spheres with a cubic-shape (voxel) discretization.

2 Code and fitted weights

The code and the fitted weights used in the present work are made available for any interested researcher to use for their needs and can be downloaded from `https://github.com/steveC91/3D-DCNN`.

References

[1] Hakon Wadell. Volume, shape, and roundness of rock particles. The Journal of Geology, 40(5):443–451, 1932.

[2] William E Lorensen and Harvey E Cline. Marching cubes: A high resolution 3d surface construction algorithm. ACM siggraph computer graphics, 21(4):163–169, 1987.

[3] Riccardo Rorato, M Arroyo, Edward Andò, and Antonio Gens. Sphericity measures of sand grains. Engineering geology, 254:43–53, 2019.

[4] Johannes Schindelin, Ignacio Arganda-Carreras, Erwin Frise, Verena Kaynig, Mark Longair, Tobias Pietzsch, Stephan Preibisch, Curtis Rueden, Stephan Saalfeld, Benjamin Schmid, et al. Fiji: an open-source platform for biological-image analysis. Nature methods, 9(7):676–682, 2012.
